# Using Performance Frontiers To Evaluate Non-OR Anesthesia (NORA) Efficiency

**DOI:** 10.1007/s10916-025-02229-5

**Published:** 2025-07-09

**Authors:** Justin S. Routman, Erik J. Zhang, Jonathan D. Blocker, Juhan Paiste, Mitchell H. Tsai

**Affiliations:** 1 Department of Anesthesiology and Perioperative Medicine, Heersink School of Medicine, University of Alabama at Birmingham, Birmingham, AL USA; 2 Department of Anesthesiology, Perioperative Care, and Pain Medicine, NYU Grossman School of Medicine, New York, NY USA; 3 Department of Anesthesiology, University of Colorado, Anschutz School of Medicine, Aurora, CO USA; 4 Department of Anesthesiology, New York Medical College, Valhalla, NY USA; 5 Departments of Orthopaedics and Rehabilitation; and Surgery, Larner College of Medicine, University of Vermont, Burlington, VT USA

**Keywords:** Non-Operating Room Anesthesia (NORA), Operational Efficiency, Performance Frontiers, Strategic Decision Making

## Abstract

**Introduction:**

In high-cost, high-revenue operating room (OR) suites, dashboards displaying key performance indicators are commonplace to optimize efficiency. Given the significant successes attained, further gains may risk compromising safety. In contrast, challenges unique to non-operating room anesthesia (NORA) sites have hindered operational efficiency. Existing productivity evaluation frameworks often fall short in guiding strategic and tactical improvements in NORA delivery. Performance frontiers have proven effective in evaluating OR systems, but their application to NORA remains unexplored. This study applies performance frontiers to assess NORA site efficiency and formulates potential operational strategies.

**Methods:**

We evaluated anesthesia billing records at our primary hospital from 1 April 2022 to 30 March 2023. Cases from operating room and NORA locations were included, except for sites with irregular volume or financial arrangements. We included only non-holiday weekdays, defining NORA block time as 7 AM to 5 PM. For each room, we calculated under-utilized (time with no anesthesia billing) and over-utilized minutes (time billed outside of NORA block hours). Data for each location were plotted as rolling 4-week sums, normalized to scheduled NORA block time. Performance frontiers were then developed and plotted.

**Results:**

Over 246 non-holiday weekdays, 42,424 cases had billable minutes during NORA block time, comprising 20,003 (47.2%) NORA cases and 22,421 (52.8%) OR cases. Performance frontiers revealed significant variability, with nonparametric tests confirming statistical significance and non-equivalence.

**Discussion:**

Performance frontiers reveal substantial efficiency variability across NORA sites, underscoring the need for targeted interventions. Some sites matched OR efficiency levels, while others showed substantial differences, particularly those with high variability and urgency. Efficient sites can leverage performance frontiers to optimize resource allocation, while inefficient locations may benefit from a shared anesthesia resource pool for real-time resource allocation. Performance frontiers provide a novel approach for operational leaders to make more effective strategic decisions.

## Introduction

Operations rooms (ORs) represent a high-cost, high revenue environment, and it is little surprise that significant resources have been allocated toward maximizing operational efficiency across the perioperative service [1]. Dashboards and scorecards displaying key performance indicators are commonplace, used to continually monitor measurements, such as case volume, throughput, and facility-specific efficiency metrics [2]. Many clinical directors and hospital administrators use the same management tactics for non-OR anesthesia (NORA) cases which offer less invasive options for medically complex patients [3]. Over the past decade, NORA volume has increased and currently comprises over one-third of total anesthesia services. Several authors have predicted that the number will soon outpace traditional OR anesthetics [4, 5].

However, NORA locations often face several unique challenges that may limit efficiency and throughput when compared to a main operating room [6]. These differences can include geographic isolation, suboptimal anesthesiologist concurrency ratios, limited interoperability, and a lack of a block allocation. At some institutions, the confluence of the latter two factors may inadvertently create potential limitations via competition for room resources by multiple NORA services [7, 8]. Further, Hudson and Lebovitz demonstrated that NORA expansion is inversely correlated with anesthesia productivity [9]. Anesthesia-specific clinical productivity metrics (e.g., total ASA units per FTE) do not apply to NORA settings because of uncontrollable factors that impact the opportunity for revenue generation, such as case volume, base units per case, and concurrency limitations [7, 9–14].

Tsai et al. have argued that performance frontiers can be utilized to compare operational efficiency among different settings, such as ambulatory surgery centers, mixed inpatient services, and NORA [15]. Plotting under- and over-utilized time to develop performance frontier curves demonstrates individual site efficiency as well as expected performance with varying resources. They allow leaders to quickly visualize operational metrics by simplifying the analysis of large datasets and potentially allowing for the monitoring of the impact of a strategic or tactical decision. This methodology is particularly useful for evaluating improvements after operational changes in OR resource allocation and comparing service lines, perhaps a more complete picture of OR metrics [1, 16, 17].

Unfortunately, due to the structural and operational differences for NORA sites, existing OR productivity frameworks are often ill-equipped to guide strategic development aimed at enhancing operational efficiency. Performance frontiers have never been systematically applied to differentiate individual NORA locations. As such, we sought to apply performance frontiers to NORA sites at a large, urban academic center in the hopes of developing a proactive operational strategy.

## Methods

At the University of Alabama at Birmingham (UAB), we extracted all anesthesia billing records during the 1 April 2022 to 30 March 2023 across all OR and NORA locations at the main campus. We included only non-holiday weekdays and defined “NORA block time” working hours from 7:00–17:00, given that NORA sites at our institution operate on fixed schedules. For each record, we captured procedure date, procedure location, anesthesia start and stop times, billed ASA base and time units, procedure type, anesthesiologist, surgeon, billing code(s), diagnoses, modifier(s), and anesthesiologist concurrency level. We used anesthesia billing time rather than operative time because the former more accurately reflected the constrained resource within NORA settings under our control. Total charges, dates of charge posting, total revenues, and dates of receipt of revenues were also included in the dataset, but they were not used for this analysis. We aggregated data by physical location rather than procedure or service line to allow for meaningful comparison of actual resource utilization between sites rather than “in-block” or “out-of-block” utilization.

For each case and location, we identified daily under-utilized time (minutes of NORA block time with no anesthesia billing) and over-utilized time (minutes of billed anesthesia time outside of NORA block time hours). For any cases that crossed midnight to or from a holiday or weekend, we considered only the portion of the case that occurred on the non-holiday weekday. Similarly, we separated minutes of each case for any that crossed either end of the NORA block time hours. We then calculated under- and over-utilized minutes using 4-week rolling sums (normalized to available NORA block time minutes) on a per room basis. We chose this method to account for any daily variability in staffing and schedules [15]. We plotted the resultant rolling sums of over-utilized time (overrun minutes) on the x-axis and the correlated rolling sums of under-utilized time (opportunity-unused minutes) were plotted on the y-axis.

We plotted performance frontiers using **R** (R Foundation for Statistical Computing, Vienna, Austria) to represent the optimal front. Each performance frontier was plotted by fitting a rational function of the form *f(x) = (a/(x-h)) +* k, where *a* represents the horizontal stretch, *h* represents the horizontal shift, and *k* represents the vertical shift graphically representing the differences in efficiency as a result of the relative distribution of under- and over-utilized time, the over-utilized time, and the under-utilized time, respectively. We repeated this process for each NORA location, the Main OR suite, and the Cardiovascular OR (CVOR) suite. With this approach, the origin coordinate at (0,0) represents optimal efficiency; the further a performance frontier is from the origin, the less efficient the represented service is. We used different colors to represent different NORA sites. Nonparametric statistical tests were used to determine statistically significant differences between performance frontiers. Data was otherwise maintained on Microsoft Excel (Microsoft Corporation, Redmond, WA). This retrospective study was approved (# 300012192) by the Institutional Review Board of the University of Alabama at Birmingham.

## Results

**Fig. 1:**
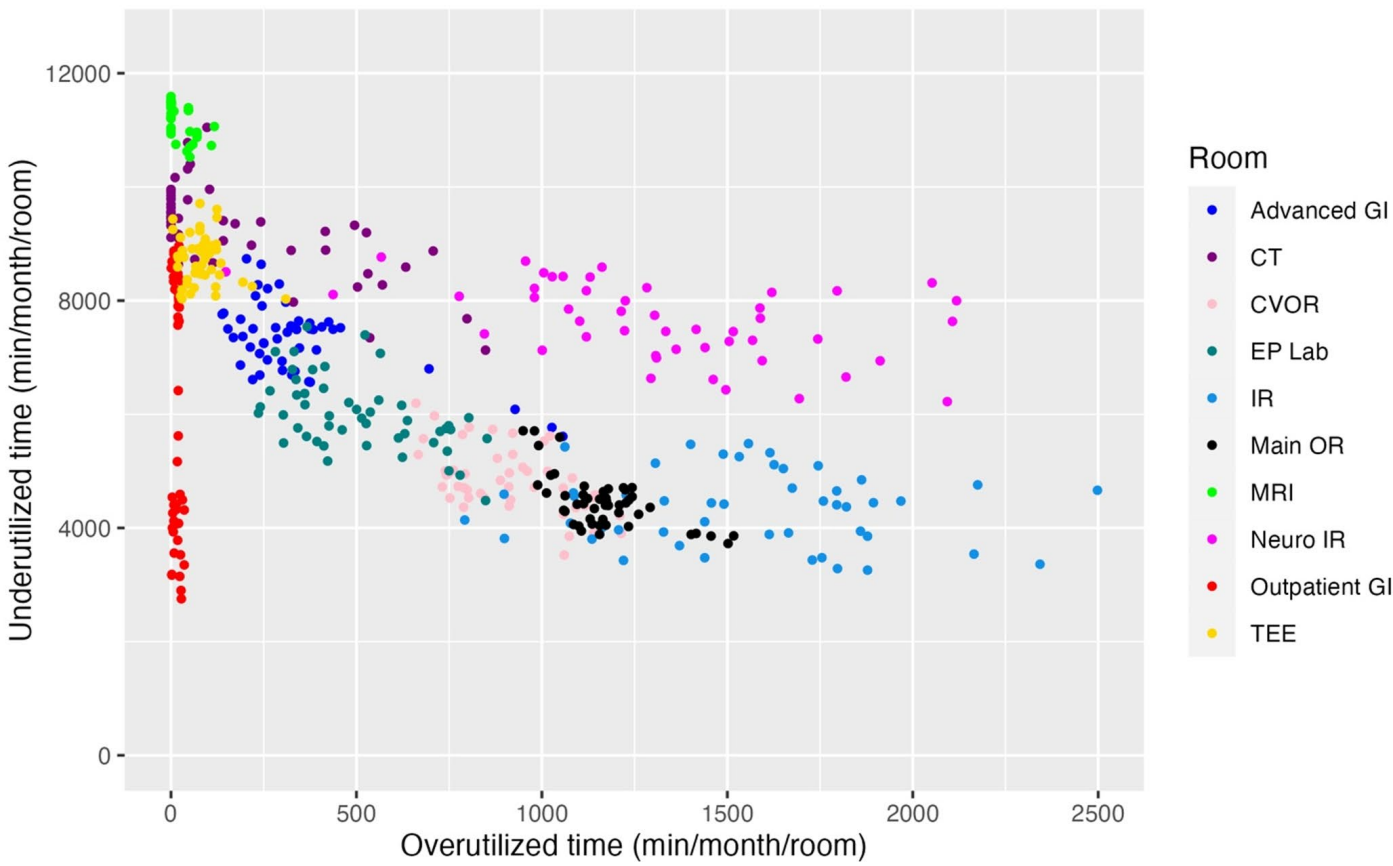
Under- and over-utilized minutes for various operating room and non-operating room anesthesia locations using normalized 4-week rolling sums on a per room basis. CT, computed tomography; CVOR, cardiovascular operating rooms; EP, electrophysiology; GI, gastroenterology; IR, interventional radiology; MRI, magnetic resonance imaging; OR, main operating rooms; TEE, transesophageal echocardiography

**Fig. 2:**
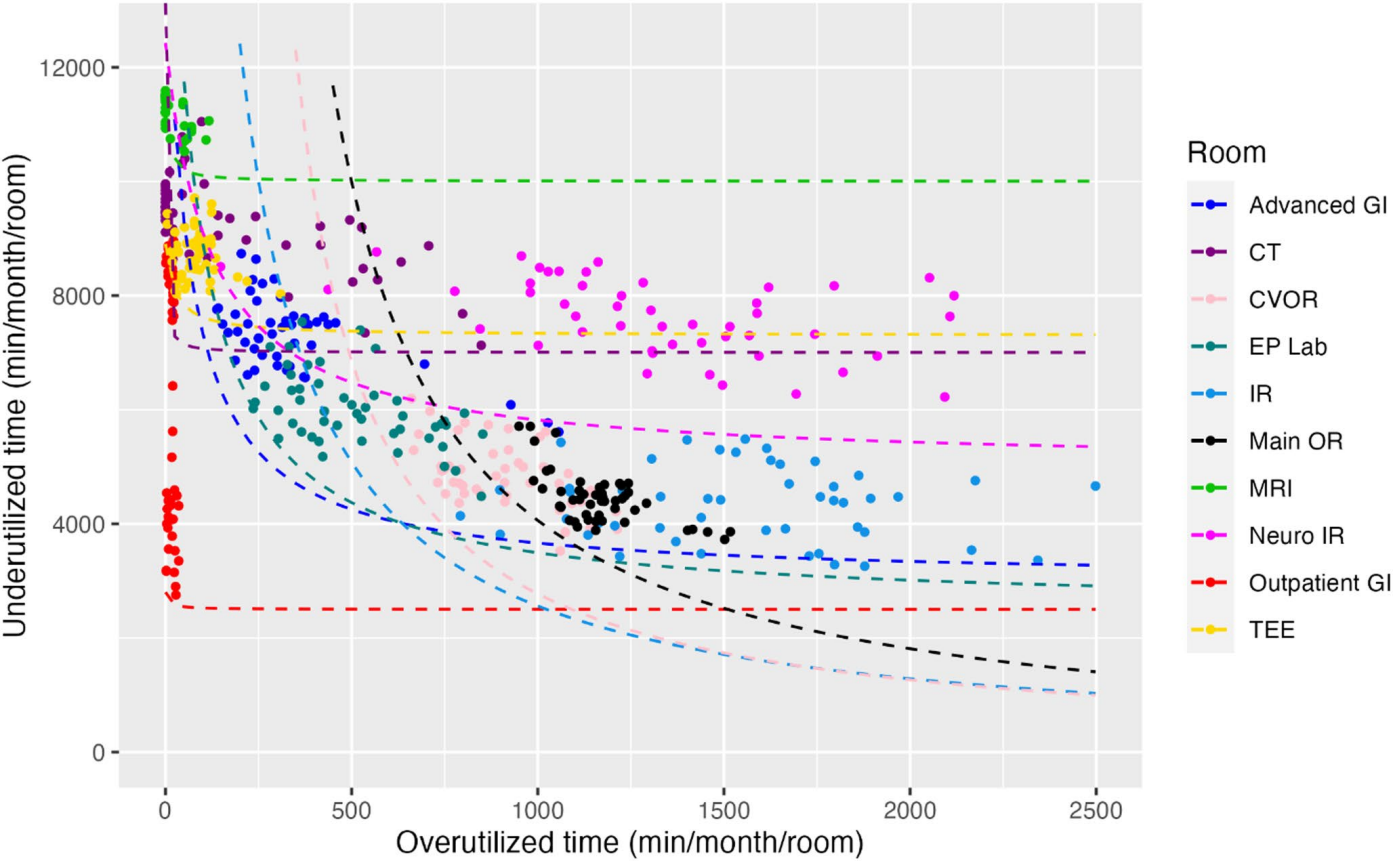
Performance frontiers added for each location plotted in Fig. 1. CT, computed tomography; CVOR, cardiovascular operating rooms; EP, electrophysiology; GI, gastroenterology; IR, interventional radiology; MRI, magnetic resonance imaging; OR, main operating rooms; TEE, transesophageal echocardiography

**Fig. 3:**
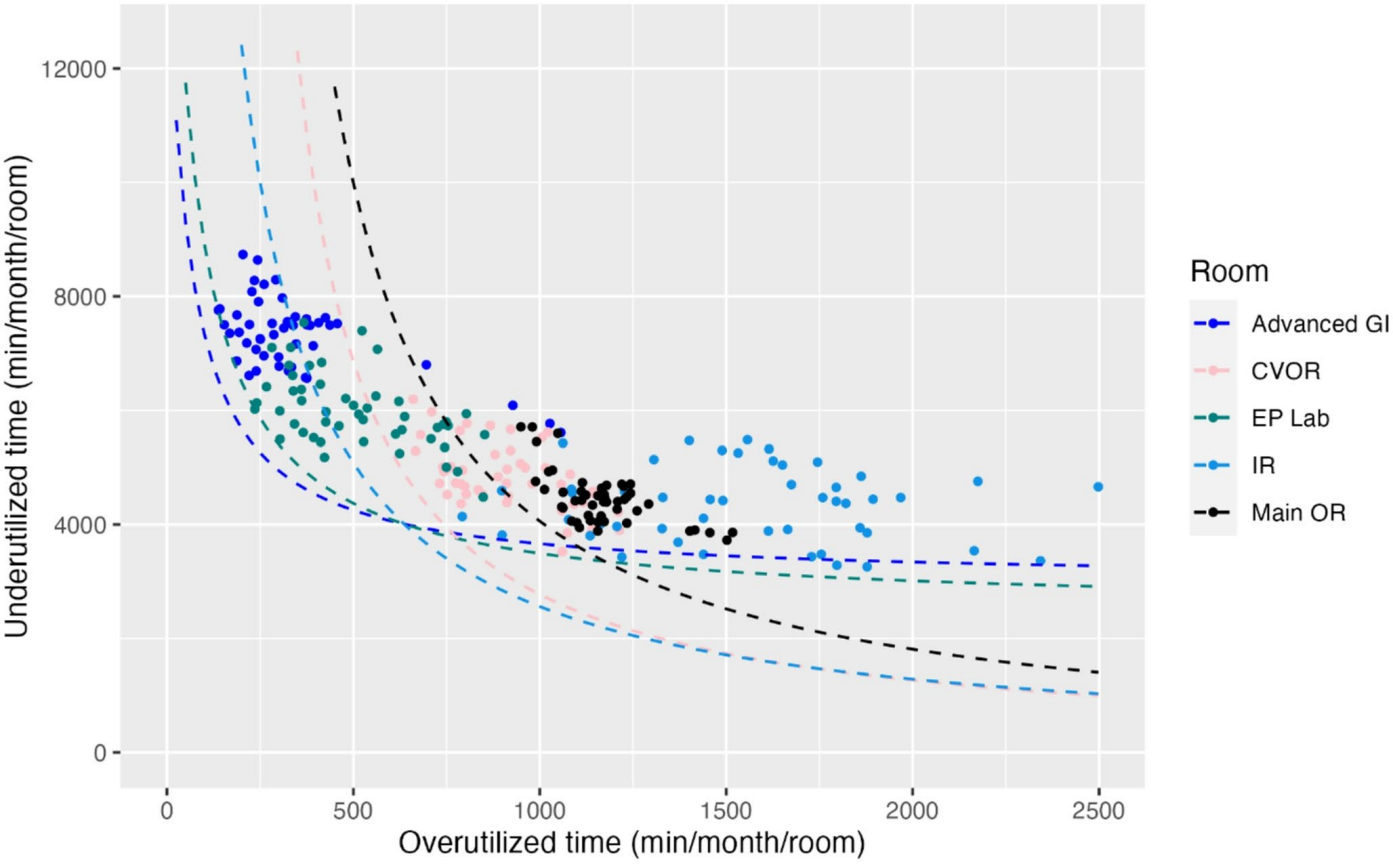
Performance frontiers of operating suites and highly efficient NORA locations only. CVOR, cardiovascular operating rooms; EP, electrophysiology; GI, gastroenterology; IR, interventional radiology; OR, main operating rooms

**Fig. 4:**
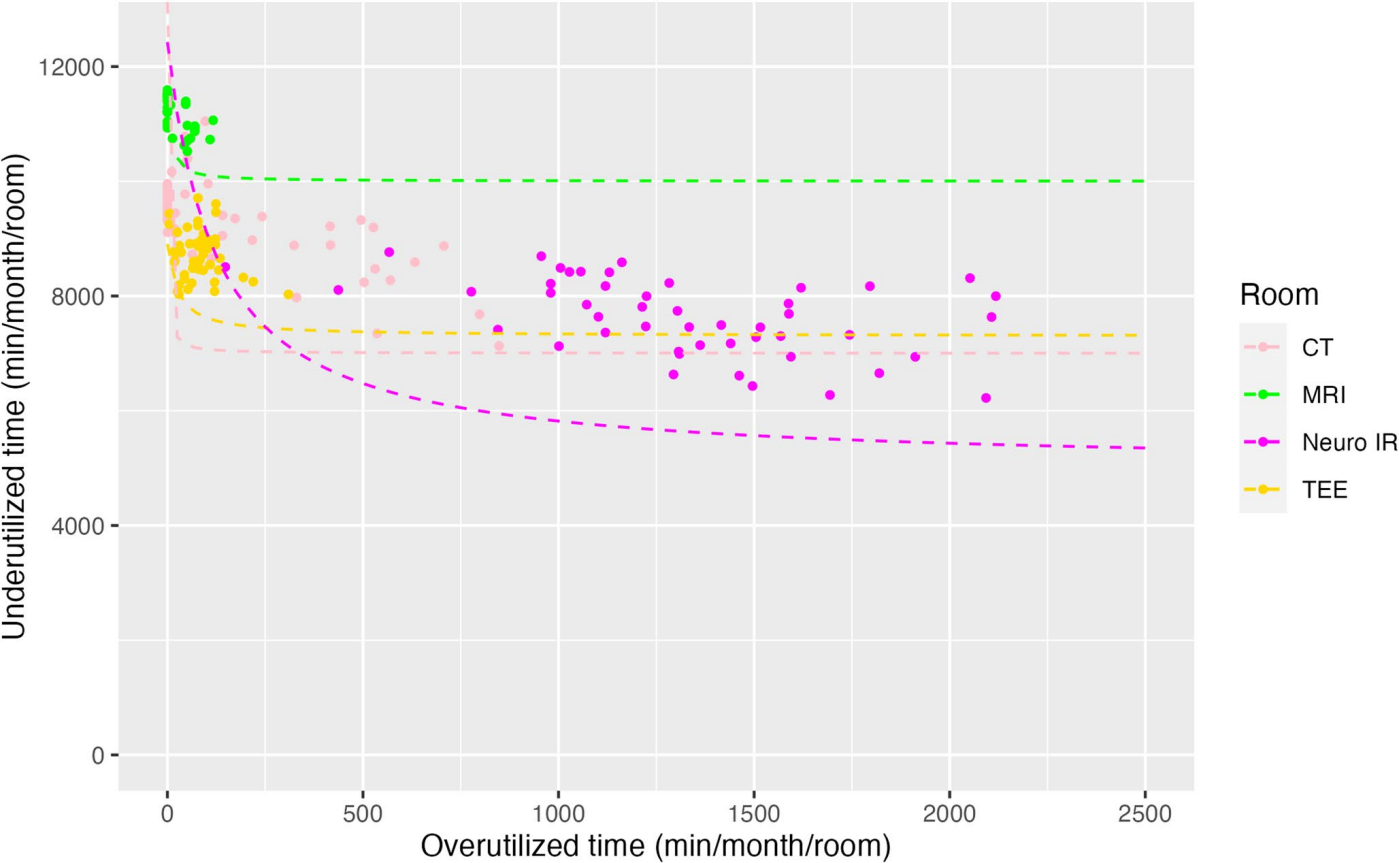
Performance frontiers of inefficient, sporadically utilized anesthesia locations consistently demonstrate a large amount of both under- and over-utilized minutes. CT, computed tomography; IR, interventional radiology; MRI, magnetic resonance imaging; TEE, transesophageal echocardiography

During the study period, there were 246 non-holiday weekdays. Records of billable anesthesia time existed for 42,424 distinct cases, of which 20,003 (47.2%) were NORA and 22,421 (52.8%) were in an OR setting. For each NORA location, we plotted under- and over-utilized time as paired 4-week rolling sums on a per-room basis, with data normalized by NORA block time availability (Fig. 1). We included data from our Main ORs (black) and CV ORs (pink) for comparison. Consistent a with previous report, [15]. we identified different groups across the NORA environment. Data for all services, with associated performance frontiers in place, is shown in Fig. 2. Kruskal-Wallis nonparametric tests comparing performance frontiers confirms the data are statistically significant and non-equivalent (*p* < 0.001). For ease of viewing and comparison, we isolated graphically similar locations and plotted them in smaller groupings. Figure 3 displays the more efficient sites, and Fig. 4 displays a subset of locations who currently receive consistent anesthesia staffing but demonstrate less consistent utilization of anesthesia resources.

For a performance frontier graph, the origin point (0,0) at the lower left corner represents the ideal state of efficiency. Increasing caseload or reducing allocated block time will shift points rightward and downward along a given curve, wherein a site is using more allocated block minutes during the designated NORA block time and/or increasing overrun beyond the end of the allocated time. In contrast, the provision of additional resources (e.g., extended block time or an additional room) shift points upward and leftward by moving case volume from the end of the day to the designated NORA block time.

## Discussion

In this study, we highlight the significant variability in efficiency across NORA locations using performance frontiers. While some NORA sites achieve efficiency similar to that of traditional OR locations, others show substantial differences, underscoring the need for targeted interventions. At our institution, NORA block times are dictated by institutional practices rather than data-based analysis of demand and efficiency. Mcintosh et al. demonstrated that appropriate block allocations are essential, and our approach enables leaders to develop site-specific strategies based on each site’s unique challenges [18]. In so doing, performance frontiers can be leveraged for strategic development and tactical decision-making.

Further, this study explores the inherent constraints and pressures imposed by these predetermined hours, revealing potential inefficiencies that arise from a lack of flexibility in operational planning. While each NORA location may request (or require) dedicated anesthesia services, multiple services are typically competing for a finite set of anesthesia resources. This constraint may limit how much operational efficiency may be improved through workflow measures used in the OR, which may negatively impact anesthesia groups and hospitals alike [7, 9]. As such, increasing NORA volumes might be a growing impediment to the financial health of both academic and private anesthesia groups. Though several strategies have been proposed to address efficiency improvement ranging from hands-off cost estimates for anesthesia subsidy negotiations to active day-before decision making, heretofore, none have shifted their viewpoint to consider the anesthesia provider as the constrained resource [19, 20].

With performance frontiers, we have demonstrated that several NORA locations at our institution have performance frontiers comparable to the main and cardiac ORs, including advanced gastroenterology (GI), electrophysiology (EP), and interventional radiology (IR) (Fig. 3). Advanced GI and EP are displaced upward and leftward along the frontier, indicating that they trend toward under-utilized time. Additional case volume (perhaps through addition of another proceduralist) or reduced anesthesia resources (e.g., less scheduled block time or an anesthesia resource shared with other NORA locations) would shift their points downward and rightward. On the other hand, IR demonstrates a high degree of over-utilized minutes. This indicates that the service might require additional anesthesia resources or that there is an inherent variability of the workload (e.g., after-hours cases). Tactically, the anesthesiology department could extend their scheduled block time or provide another block allocation during daytime work hours, recognizing that there might be a net loss of operational efficiency for anesthesia providers.

In contrast, inefficient, sporadically utilized locations consistently demonstrate both under- and over-utilized minutes, as shown in Fig. 4. Similarly, Schottel and colleagues showed that trauma services experience significant variability in workload and consistently face inefficiencies with both under- and over-utilized minutes [17]. This inefficiency underscores the challenges of managing capacity-based services using traditional metrics. Neurointerventional radiology is the archetypal example at our institution, given that emergent stroke thrombectomies occur at any time of day and nearly always require anesthesia services. This sporadic resource utilization is reflected graphically in Fig. 4, where its performance frontier curve is much farther from the origin than those of more efficient sites in Fig. 3. At an operational level, most clinical directors simply reconfigure the staffing available to provide anesthesia coverage on an ad hoc basis.

Implementing a shared “sandbox” approach allows for flexibility and adaptation based on demand and operational dynamics, optimizing real-time resource allocation. A flexible, shared scheduling system has been previously shown to improve utilization of both elective time-in-block as well as opportunity-unused time, accommodating increased case volumes without additional resources [21]. Varied operating hours stem from several factors, such as patient volume, equipment considerations, location oversight philosophy, lack of block time, limited interoperability due to room/equipment specialization, emergency procedures, and staffing patterns. A dynamic resource allocation model better addresses the challenges of capacity-based services by shifting from rigid block allocations to a system that reallocates resources based on real-time demand. Such on-demand staffing necessarily requires a certain amount of slack in the system for emergencies, but anesthesia departments should not be left subsidizing this inherent inefficiency. Presumably, performance frontiers can justify consolidation of resources, the expansion of anesthesia and nursing coverage, or even the institutional support necessary for structural investments.

As a final example, an ambulatory center-like cluster is also noted hugging the y-axis in Figs. 1 and 2, demonstrating significant amounts of under-utilized time, consistent with previously reported data [15]. Ambulatory surgical centers mostly cover elective, outpatient surgeries, and the variability in both case length and hours is less variable than mixed patient settings. Here, the hospital administrators can increase case volume, reduce the number of rooms (e.g., from 6 to 4), or shorten block allocations. For anesthesia services, staff scheduling considerations should include concurrencies, case complexity, regional anesthesia demands, and post-operative care unit coverage.

While performance frontiers highlight whether a site maps toward one end of the spectrum, they do not diagnose the root causes behind these patterns (e.g., sterile processing delays, turnover bottlenecks, or other factors that may initially seem unrelated to anesthesia staffing). However, they can be used to quickly pinpoint these misalignments and guide targeted interventions, even when the root causes themselves lie outside the direct control of anesthesia services. For example, if under-utilized time is driven by sterile processing delays and over-utilized time is also present, resolving the equipment delays would reduce both inefficiencies, shifting data points toward the origin (0,0). Alternatively, anesthesia groups can adapt their staffing models, decoupling their schedule from procedural block time, flexing in and out of rooms “on demand” rather than providing continuous coverage. This staffing model would minimize idle time while still meeting demand, thereby moving the data points closer to the origin. If under-utilized time is driven by delays, but over-utilized time is minimal, fixing those delays may shift under-utilized time to the end of the workday—highlighting a new opportunity to reduce block time, adjust staffing, or add additional surgical cases. These insights illustrate how performance frontier data can be used iteratively to address broader system issues and refine local strategies for resource allocation and operational improvement. Table 1 illustrates how performance frontier data can inform context-specific interventions; examples shown are hypothetical and not intended to be comprehensive or prescriptive.

**Table 1:** Representative scenarios, strategies, and anticipated effects based on observed under- and over-utilized time patterns in performance frontier analysis. Examples shown are hypothetical and not intended to be comprehensive or prescriptive

Utilization Pattern	Over-Utilized Time Present	Over-Utilized Time Not Present
Under-Utilized Time Present	*Scenario*: Mid-day delays (e.g., turnover or equipment issues) create idle time, pushing cases beyond block end. *Strategy*: Address process inefficiencies to reduce intra-day delays. *Effect*: Compresses the schedule and allows the day to finish earlier, reducing both under- and over-utilized time and shifting the data point toward the origin.	*Scenario*: Mid-day delays (e.g., turnover or equipment issues) create idle time, though low case volume means cases still finish early. *Strategy*: Resolve delays, then reevaluate and adjust block time or staffing; consider reducing coverage or adding cases if possible. *Effect*: Fixing delays may initially increase idle time, highlighting excess resources; subsequent staffing reductions shift data points back toward the origin.
Under-Utilized Time Not Present	*Scenario*: High case demand exceeds allocated time causing consistent late finishes; no significant intra-day idle time. *Strategy*: Add a second room or extend hours; coverage can be staggered and uneven. *Effect*: Longer block or decanting cases over addidtional rooms reduces overutilized time, shifting data point closer to origin.	*Scenario*: Current resources are well-aligned with case volume and flow. Minimal idle or overtime. *Strategy*: Monitor regularly; use as internal benchmark. *Effect*: Data point remains close to origin.

**Fig. 5:**
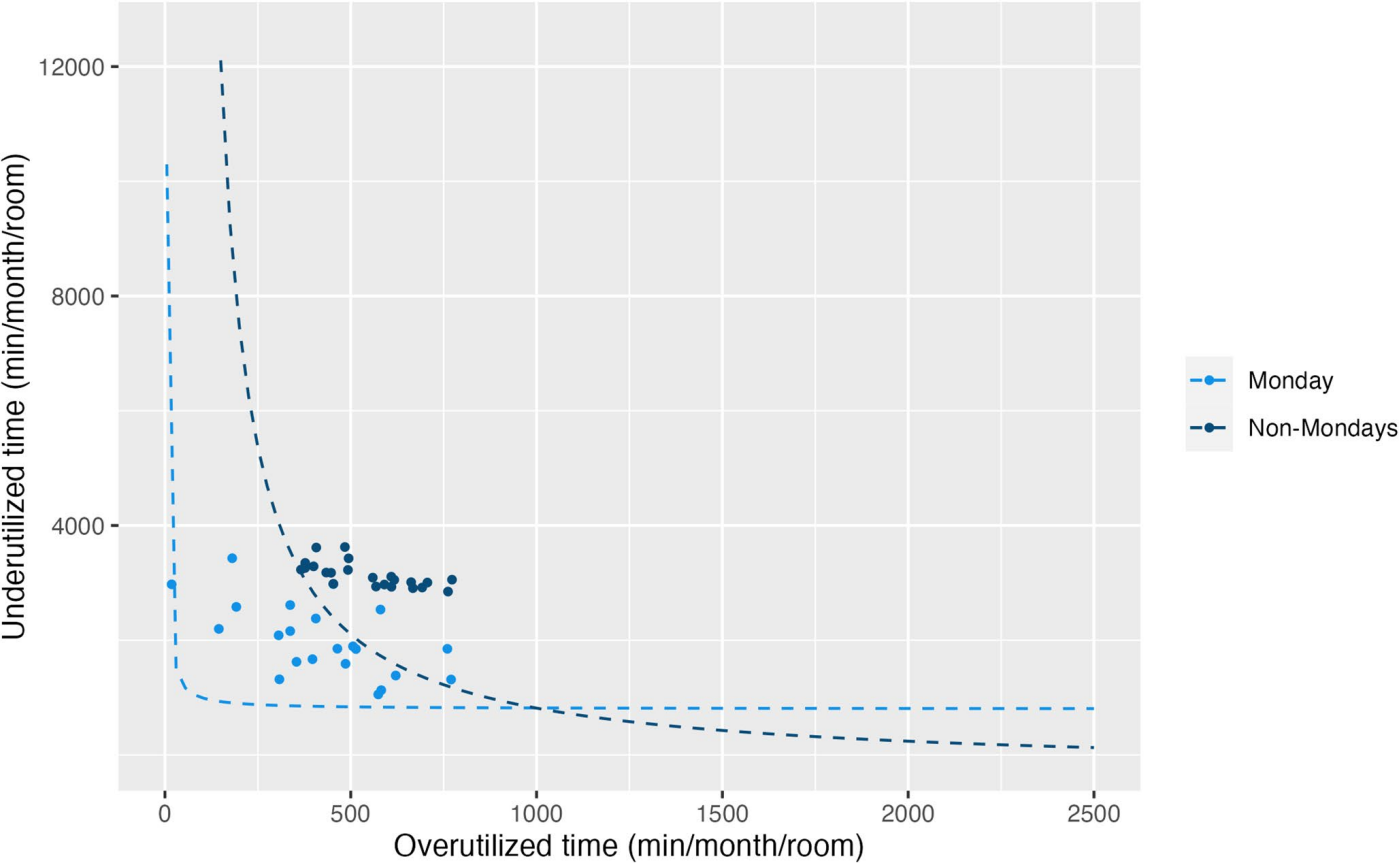
Performance frontier curves for interventional radiology (IR) cases on Mondays versus non-Mondays following the addition of a second anesthesia-covered IR room one day per week

Although no formal operational changes have been implemented in response to our performance frontier analysis, its development coincided with a request for expanded anesthesia support by the interventional radiology service. Informed in part by insights from our analysis, departmental leaders piloted a second anesthesia-covered IR room one day per week (Mondays). This provided a natural opportunity to apply the framework retrospectively. Using anesthesia billing data from non-holiday weekdays between 1 November 2024 and 30 April 2025, we compared frontier curves for Mondays versus non-Mondays, again assuming 10-hour primetime blocks, normalized by number of days. The Monday PF curve was significantly closer to the origin, reflecting significant reductions in both under-utilized (*p* < 0.01) and over-utilized (*p* = 0.02) time (Fig. 5). Case volume data for the period (125 cases on Mondays, averaging 4.8 cases per day, vs. 362 cases on non-Mondays, averaging 2.8 cases per day) suggest that while some volume was new, much was likely redistributed from end-of-day overages into added prime-time capacity. While unmeasured differences between Monday and non-Monday scheduling may limit interpretation, this result retrospectively supports the performance frontier-informed strategy outlined in Table 1 and demonstrates improved alignment between anesthesia resources and procedural demand.

There are several limitations to the present analysis. First, all data comes from a single academic medical center’s primary hospital and the results may not be generalizable to other locations. Several authors have identified significant productivity differences between and within anesthesia groups across academic and private practice settings [10, 13]. The specific frontier shapes presented here should not be interpreted as benchmarks, but rather as exemplars of the methodology. Sites with highly irregular financing models or extremely low case volume (e.g., fewer than 1 case per day) were excluded from analysis, which may further limit generalizability. Second, the present study does not distinguish any difference in cost between under- and over-utilized time. Since portions of fixed costs for “prime” time teams may differ from those covering end of day and overnight work, strategies to improving a given site’s operational efficiency must balance the clinical workload and costs.

While it may be enticing to develop cross-institutional benchmarking of NORA performance frontiers, defining a universal frontier is currently impractical. Doing so would require industry-wide definitions and implementations of block time, as well as adjustments for local case-mix, payor mix, staffing ratios, and regional labor/overhead costs, all of which vary markedly among hospitals. The primary strength of this approach therefore lies in intra-institutional use, allowing each site to function as its own control while also permitting rapid, locally tailored iteration of staffing or scheduling changes. Future studies incorporating direct costs and revenues may facilitate more meaningful inter-institutional comparisons. While performance frontiers cannot overcome broader system-level inefficiencies, they nonetheless provide a pragmatic, site-specific framework to guide incremental improvements in resource allocation within each institution’s unique environment.

Our study utilized 7:00 AM until 5:00 PM on weekdays as “NORA block time” and block allocations may vary across institutions. Not only do these hours not represent typical anesthesiologist working hours nationwide, but they may also not represent the typical operating hours of certain NORA sites. At our institution, these NORA location hours are effectively set by hospital policy, aligning with nursing shifts rather than data-driven optimization. When expanding anesthesia coverage to new NORA service lines, we have previously established full-day coverage up front, anticipating that case volumes will quickly grow to match capacity. However, this anticipated growth may not materialize, resulting in coverage models that exceed actual clinical need. Such fixed scheduling impacts resource utilization, underscoring the need for performance frontiers to periodically evaluate and recalibrate allocation. This adaptable methodology can similarly be tailored to meet the needs of other individual sites or institutions as well.

For this analysis, we chose to use anesthesia billing time as our measure of resource utilization rather than procedure time. While procedure time uses the proceduralist as the constrained resource and room occupancy time centers on the room itself, using anesthesia billing time highlights the anesthesia provider as the limiting factor. This approach not only captures the full scope of provider utilization—encompassing all pre- and post-procedure patient-related activities—but also allows us to adjust based on actual demand. Unlike proceduralist or room availability, which are beyond our control, the staffing of anesthesia providers can be adjusted to align with trends in utilization. Our approach assumes that the most efficient systems will plan and conduct their operative work during allocated times and conduct no work during non-allocated times. Despite these potential shortcomings, our study effectively demonstrates the utility of applying performance frontier methodology to the NORA setting, providing a baseline against which to evaluate subsequent successes or failures.

In closing, clinical operations teams can avoid the difficulties of comparing productivity across different sites or against national standards simply by using their own service as the comparator. Future studies employing performance frontiers can extrapolate beyond the current metrics, further highlighting the gap between the present state and aspirational benchmarks. By facilitating strategic planning and continuous assessment of tactical and operational decisions, perioperative services can leverage performance frontiers to optimize operations, improve efficiency and procedural timeliness, and perhaps, increase profitability.

## Data Availability

No datasets were generated or analysed during the current study.

## Abbreviations

ASA: American Society of Anesthesiologists
CT: Computed tomography
CVOR: Cardiovascular operating rooms
EP: Electrophysiology
FTE: Full-time equivalent
GI: Gastroenterology
IR: Interventional radiology
MRI: Magnetic resonance imaging
Neuro IR: Neurointerventional radiology
NORA: Non-operating room anesthesia
OR: Operating room (or main operating rooms, when indicated)
TEE: Transesophageal echocardiography
UAB: University of Alabama at Birmingham
